# Medical and Physical *Intelligence*

**Published:** 1800-02

**Authors:** 


### MEDICAL AND PHYSICAL *INTELLIGENCE*,

### ( Original and Selected. )

CIT. HALLE read a Memoir on the *History of the Curative Art*, in which he particularly considered the periods when it has made real progress. He states the first epoch to have been from the time of Hippocrates to that of Sanctorius; the second, at the time of Sanctorius; as he was the first who boldly appealed from the doctrine of the ancients to experience; the third, at the restoration of the physical sciences in Europe; and the fourth, from the discovery of various aëriform fluids, and the introduction of modern chemistry. He inquires, during each of these periods, into the state of the curative art, or the art of preserving the health of man in society, either by its connection with the legislature, the manners of the age, or the public police; as, likewise, the art of preserving the health of man considered as an individual.

This Memoir contains some valuable remarks on physical education, gymnastic exercises, dress, philanthropic establishments; others, for the prevention of contagious maladies; on houses of correction; on the diseases of soldiers; on the influence which the construction of towns and villages, as well as their situation, manifest on the health of citizens and husbandmen. The substance of this paper has been inserted in the French Methodical Encyclopedia, under the head of *Hygiène*.—*Rapport Géneral des Travaux de la Société Philomathique*, Vol. II. p. 81, 82.

---

Citizens ADET and CHAPTAL presented Memoirs on the *difference between the acetic and acetous acids*. The former, in consequence of repeated experiments, was of opinion that these two acids did not differ on account of their proportions of oxygen, but by a greater degree of concentration which the acetic acid owes to the evaporation of its water, in a combination with metallic oxyds, or alkalies. He made an unsuccessful attempt to superoxygenate the acetous acid, or to disoxygenate the acetic acid; and he hoped eventually to ascertain the non-existence of the acetous, and that the only difference between the acetic acid extracted from the acetat of copper, and that from vinegar, depended on the smaller quantity of water contained in the former. Cit. Chaptal has drawn different conclusions from his experiments. He always discovered in the acetic acid, though it possessed only the same degree gree of concentration as the acetous acid, a more penetrating fmell and tafte, and that it was much more powerful in the diffolution of earths and metallic oxyds. New experiments have induced him to believe, that the difference between thefe two acids muft be afcribed to the circumftance, that the acetic acid contains a fmaller quantity of carbon than the acetous.—*Ibid.* p. 25, 26.

---

Cit. VAUQUELIN communicated to the Society, two Memoirs, on a *new metallic acid* which he difcovered in the red lead of Siberia. He afcertained, by experiments, that this fubftance differed from all other known minerals; as it had the property of changing all its faline or earthy combinations to a red or orange colour. This property, and that of producing variegated and beautiful colours when combined with metals, induced him to give it the name of *chrôme.* Its reduction is effected by carbon only, in a ftrong fire; and on treating it with nitric acid, the chromic acid is reproduced. The chrôme abforbs two thirds of its weight of oxygen, in order to become an acid. This acid might furnifh painters with an enamel of permanent colours. Cit. Vauquelin has fince met with chrôme in a natural ftate, in a green oxyd of lead, and in feveral other fubftances.—*Ibid.* p. 26, 27.

---

Cit. VAUQUELIN communicated to the Society, an *analyfis of the ruby,* in which he found the chrômic acid of Siberian red lead in a proportion of $4 \frac{7}{10}$; but he could not difcover the Silex mentioned by KLAPROTH; hence he concluded that the ruby is a kind of faline combination of chrômic acid and alumine. — *Ibid.* p. 27.

---

The fame member announced that he had difcovered *chrôme and a new earth in the emerald,* and that he had formerly found thefe fubftances in the beryl. He alfo prefented the analyfis of the pulverulent chlorite, which contained 26 parts of filex, $18 \frac{50}{100}$ of alumine, 8 of magnefia, 43 of oxyd of iron, 2 of muriate of foda, or potafs, and 2 of water; a refult which is different from all that have hitherto been given in the analyfis of chlorites. He attributes this difference, rather to the nature of this apparently mixed earth than to the inaccuracy of chemical operations.—*Ibid.* p. 28, 29.

---

He alfo prefented the refult of his inquiries *on the extractive principle of vegetables,* which has hitherto been but imperfectly known. A number of experiments made on the extracts have convinced him, that they are of a very compound nature. He found that among the faline fubftances contained in the extract, properly called the acetous acid, the acetites of potafs, lime, and ammonia, are thofe only which are conftantly difcoverable; that the extract is compofed of carbon, hydrogen, oxygen, and azote; and that its property of attracting the humidity of the air, as well as moft of its its medical virtues, are principally owing to the preſence of the acetat of potaſs.—*Ibid.* p. 29.

---

The ſame member, in conjunction with Cit. Fourcroy, analyſed a *gouty concretion* which had been ſpontaneouſly formed *in the fingers of a man,* that were ſwelled in the form of a large pear, while his limbs were entirely diſtorted by that diſeaſe. They found that this ſubſtance was the urat of ſoda combined with a conſiderable proportion of animal matter. The authors, however, did juſtice to M. Tennant, by acknowledging that he had previouſly diſcovered, that arthritic concretions were a combination of lithic, or uric, acid and ſoda.—*Ibid.* p. 30, 31.

---

Cit. Fourcroy, while employed in this important reſearch, has written a Memoir on the *Chemical and Medical Hiſtory of Human Urine,* in which he has deſcribed all the ſaline ſubſtances contained in this excretion. He obſerved, that there exiſted a particular ſubſtance, twenty times more conſiderable than all others combined, and which merited a particular inveſtigation, the reſult of which he propoſed to communicate to the Society, at a future period.—p. 32.

---

Cit. Lasterie read a Memoir on the manner of fabricating *alcazarras,* or *vaſes of a very porous earth,* uſed in Spain for the purification of water. Theſe vaſes are compoſed of about one-third of calcareous earth, one-third of alumine, one-third of ſilex, and a very ſmall proportion of iron. The compound earth is divided, tempered, mixed with a twentieth part of ſea-ſalt, and when it has acquired a ſufficient conſiſtence, it is put into the furnace; after which the vaſes formed of it are half baked. Their poroſity muſt be aſcribed to this proceſs, and the addition of ſea-ſalt. The water kept in theſe veſſels is gradually filtrated through their pores, and by its continual evaporation acquires a conſiderable degree of cold. They are much uſed in Spain and Portugal, where they were introduced by the Arabs; they are likewiſe made uſe of in the Weſt Indies, and ſome parts of Africa. The earth proper for making them is common in France, and the various economical uſes to which they might be applied, renders them an important object of manufacture.—*Ibid.* p. 32, 33.

---

In the courſe of the year 1798, there were eight hundred and thirty-three *patients* received into the *Royal Hoſpital of Stockholm,* including forty-five who remained from the preceding year. Of this number, five hundred and fifty-ſix have been cured; one hundred and five died, eight were incurable, and ſeventy-nine remained for cure. Among theſe patients, the aſtoniſhing number of two hundred and thirty were afflicted with venereal complaints! one hundred and ſeventy-two with different kinds of fever; ſeventy-five caſes of dropſy, twenty-five of phthiſis, &c.—*Le Nord Littéraire,* No. viii. p. 359.

From

From the laft examination of the Regifters belonging to the *Houfe of Education at Stockholm,* it appears, that out of 1227 children who have been admitted, 1026 have died; being nearly five out of fix. This enormous mortality, which fo frequently attacks children in the firft year of their age, has engaged the attention of a philanthropift, who has endeavoured to inveftigate the caufe of this afflicting and increafing mortality. *Ibid.* p. 360.

---

M. Rafn lately read to the Academy of Sciences at Copenhagen, an Effay on fome experiments made by him three years ago, on the vegetation of plants, in feveral mixtures of different forts of earth; refearches which he propofes to continue. It appears from thefe experiments, that it is principally carbon which contributes to the nutrition of the plants, and the fertilization of the foil.—*Ibid.* p. 371.

---

### *Excellent Writing Ink.*

A feries of experiments made by Ribancourt, has furnifhed us with the following valuable receipt for producing an uniformly black and permanent ink. Eight ounces of galls and four of logwood are boiled with twelve pounds of water for an hour, or till one half of its quantity is evaporated. This liquor is then percolated through a hair fieve, and four ounces of vitriol, or fulphat of iron, three ounces of gum arabic, one ounce of copperas, or fulphat of copper, and one ounce of fugar-candy are added. The whole mafs is ftirred to promote the folution of the falts and gum, after which it is left to ftand for twenty-four hours. The liquid is then poured off from its coarfe fediment, and preferved in well ftopped glafs, or ftone jars. This ink acquires a beautiful black colour, which it retains for a long time.—*Chem. Annal.* Vol. II. p. 48.

---

### *Domeftic Intelligence.*

Institution for the Inoculation of the Vaccine-Pock, at No. 36, Warwick-ftreet, Golden-fquare. Founded Dec. 2, 1799.

THOSE who are acquainted with only a part of the hiftory of the Small-Pox, fcarcely take into their contemplation more than the advantages of the *inoculated* over the *natural Small-Pox,* in the points of prefervation of the lives of individuals, and the fubftitution of a difeafe generally flight for a difeafe generally fevere; and fuch perfons imagine, that the practice of Inoculation neither requires, nor is, perhaps, capable of farther improvement: But thofe who are more extenfively acquainted with the hiftory of the Small-Pox, know that it is productive of a great deal of mifchief, notwithftanding the advantages of Inoculation—for,

1. Under

1. Under the beſt treatment, a certain proportion of perſons die in the inoculated Small-Pox; and although the proportion of deaths to the recoveries may not exceed Five out of a Thouſand Patients, the diſtreſs occaſioned by theſe fatal caſes is more ſeverely felt than when ſuch caſes occur in the caſual diſeaſe: therefore the ſubſtitution of a milder diſeaſe will contribute to leſſen the diſtreſs which would be thereby occaſioned.

2. It ſeems fair to calculate, that, in the inoculated Small-Pox, one in twenty five patients undergoes a ſevere diſeaſe.

3. The numerous ſources of the Small-Pox infection now preclude every proſpect of extinguiſhing this diſeaſe: and unleſs Inoculation were univerſally practiſed, it is moſt likely that the proportional mortality by the natural Small-Pox is rather increaſed than diminiſhed, in conſequence of the more extenſive diſſemination of the infection by Inoculation.

4. In a certain proportion of inoculated caſes of Small-Pox, deformities of the ſkin are produced, which no practitioner can be anſwerable for preventing in any inſtance. Diſeaſes are alſo frequently excited by inoculation, to which a diſpoſition pre-exiſted in the conſtitution.

5. In particular families, and in particular ſtates of the conſtitution, as in pregnancy, &c. the Small-Pox is an exceedingly dangerous diſeaſe, even by inoculation. Now, it is manifeſt, from the accounts which have been collected of the diſorder called by the name of the Cow-Pock, and particularly from the experience by inoculation of it ſince January laſt, that the hurtful effects of the Small-Pox above ſtated may be prevented, by ſubſtituting for it the inoculation of the Cow-Pock—Becauſe,

1. Of above four thouſand perſons who have had the inoculated Cow-Pock, one only has died. There is, however, good ground for believing, that the proportional mortality will be even leſs than here ſtated.

2. Not a ſingle well-atteſted inſtance has been produced, among more than 2,000 of the above perſons, known to have had the inoculated Vaccine Pock, and who were ſubſequently inoculated for the Small-Pox, of this diſeaſe being ſubſequently taken; although many of theſe were alſo expoſed to the infectious effluvia of the natural Small-Pox. And, traditionally, this fact has been eſtabliſhed time immemorial, with regard to the caſual Cow-Pock.

3. It may ſafely be affirmed, that the inoculated Cow-Pock is generally a much ſlighter diſeaſe than the inoculated Small-Pox; and that the proportion of ſevere caſes in the latter is to the former as at leaſt ten to one.

4. It does not appear that the genuine Vaccine Pock can be propagated like the Small-Pox, by effluvia from perſons labouring under it. Hence, if the Vaccine Inoculation ſhould be univerſally inſtituted in place of the Small-Pox, it is reaſonable to conclude, that this moſt loathſome and fatal malady will be extinguiſhed; and, like the Sweating Sickneſs, Plague, certain kinds of Leproſy, &c. be known in this country only by name.

5. It

5. It does not appear that the Vaccine Poifon, like that of the Small-Pox, can be conveyed fo as to produce the difeafe indirectly from difeafed perfons, by adhering to clothes, furniture, bedding, letters, &c. Hence no danger of its propagation in thefe channels is to be apprehended from the univerfal practice of the Inoculation of the Cow-Pock.

6. It has been found that a perfon, whofe conftitution has diftinctly undergone the Vaccine difeafe, is in future unfufceptible of the fame diforder. Hence no objection can be made to the new Inoculation, as was once urged, on account of its being believed, that, by the commutation of the Small-Pox for the Vaccine Pock, an eruptive difeafe would be introduced, to which the fame perfon would be repeatedly liable.

7. It does not appear that thofe who have already gone through the Small-Pox are fufceptible of the Vaccine difeafe, as was a little time ago believed. Hence no objection can be urged on the fcore of perfons who have already gone through the Small-Pox being liable to a new infectious difeafe, by the introduction of the Vaccine Inoculation.

8. Experience fhews, that there is no reafon to apprehend the fmalleft chance of deformities of the fkin from the Vaccine Inoculation.

9. The extenfive practice of the Vaccine Inoculation in the prefent year, and the accounts of the difeafe in the cafual way, do not fhow that any other difeafe will be excited fubfequently, which is peculiarly imputable to the new practice.

It may be ufeful to add, that the prefent Inftitution is perhaps the beft imaginable for procuring evidence to inform thofe who are unacquainted with the new practice; for determining all doubtful points relating to it; and for difcovering errors: as every cafe will be regiftered; every new trial be made under the direction of the Medical Eftablifhment belonging to the Inftitution; and the refults of the practice will be reported to the Governors.

From the above comparative ftatement, it is manifeft that it is highly to the intereft of the Britifh Public to adopt univerfally the Inoculation of the Vaccine Pock in place of the Small-Pox. And that the pooreft ranks in fociety may enjoy the benefit of the new Inoculation, the following Plan of an Inftitution is fubmitted to the confideration of benevolent perfons; confiding, that it will be readily perceived, that, perhaps, no charitable inftitution ever promifed to be productive of fo much benefit at fo little expence; and that, when the objects are well underftood, it will receive fuch aids as are neceffary to its eftablifhment and maintenance.

The predeceffors of our auguft Sovereign afforded a decifive proof of their wifdom and philanthropy in introducing the Small-Pox Inoculation, not only by the encouragement which they gave to others, but by fetting the example of the practice in their own family: and a moft diftinguifhed Prince of the fame illuftrious family, having previoufly not thought it unworthy of him to be informed, by the experience of the new Inoculation, among certain orders in society, to whom he graciously extends protection, has deigned to confer the honour of his Patronage on the present Institution.

### PLAN.

1. At a House to be called The INSTITUTION FOR THE COW-POCK or VACCINE INOCULATION, a Physician and a Surgeon shall attend every Tuesday and Friday, at one o'clock, to examine, inoculate, and prescribe for the Patients, who shall attend at the Institution at such times as they shall be directed by the Physician and Surgeon.

2. An Apothecary shall also attend at the same time with the Physician and Surgeon, to discharge the duties of his department.

3. The Patients admitted to receive the benefits of the Institution shall be those who apply with recommendatory letters from the Governors.

4. The Patients shall be supplied with proper medicines at the expence of the Institution, and, when necessary, be attended at their own houses.

5. Subscribers of one guinea annually to the Institution shall be entitled to a right of having two Patients constantly on the books of the Charity; or they shall have the same right during life, by paying ten guineas at one time. Subscribers of larger sums may have the right of having a proportionally greater number of Patients constantly on the books.

6. The Subscribers are to be called Governors; they shall possess the power of transacting all the business relating to the management of the Institution, in such a manner as shall be agreed upon by themselves.

7. The Subscriptions shall be employed to defray the expences of the Institution.

8. The establishment belonging to the Institution shall consist of a Patron, a President, six Vice-Presidents, a Treasurer, and the Governors, besides the necessary Medical Officers for carrying on the business which is the object of it.

9. The medical duties are to be discharged gratuitously by two Physicians, two consulting Surgeons, two Surgeons, and three visiting Apothecaries. These officers are to be Governors.

10. There shall be a resident Apothecary, to prepare and dispense medicines; a Secretary, a Collector, a Porter, and such other Officers as shall be found necessary.

The Form of a recommendatory Letter:

*I recommend the Bearer,* *as a proper object for the Benefit of Inoculation at the Cow-Pock Institution.*

PATRON,

His Royal Highness the Duke of York.

PRESIDENT,

VICE

VICE PRESIDENTS,

| | |
|---|---|
| Right Honourable Lord Petre | Sir William Lee, Bart. |
| Sir George Baker, Bart. | H. J. De Salis, D.D. F.R.S. |
| William Devaynes, Efq. M.P. | William Adam, Efq. M.P. |

TREASURER,

Stephen Aifley, Efq.

PHYSICIANS,

George Pearfon, M.D. F.R S. | Lawrence Nihell, M.D.

CONSULTING SURGEONS,

Thomas Keate, Efq. F.R.S. | John Rufh, Efq.

SURGEONS,

Robert Keate, Efq. | John Gunning, Efq.

VISITING APOTHECARIES,

Auguftus Brande, Efq. | Francis Rivers, Efq. | Mr. Everard Brande,

RESIDENT APOTHECARY,

Mr. J. Lewis.

A printed Account of the Inftitution may be had, and Subfcriptions are received at the Inftitution, No. 36, Warwick Street; and at the banking houfes of Devaynes, Daws, and Noble, 39, Pall Mall; Coutts and Co. 59, Strand; Ranfom, Morland and Co. 57, Pall Mall; Hammerfleys, Montolieu, and Greenwood, Pall Mall; Birch, Chambers and Hobbs, New Bond Street; Edwards, Templer, Middleton, Johnfon and Wedgewood, 18, Stratford Place, Oxford Street; Wright, Selby, and Robinfon, 5, Henrietta Street; Child and Co. 1, Fleet Street; Prefcot, Grote, and Prefcot, 62, Threadneedle Street.

---

Dr. Pearfon will commence his ufual fpring courfes of Lectures on the Materia Medica, Practice of Phyfic and Chemiftry, on Monday, February 3d, at his Elaboratory, in Whitcomb-ftreet, Leicefter-Square.

---

Dr. Crichton's Medical and Chemical Lectures, (which by accident have not been announced in our laft Number) commenced on Monday, January 27th, at his houfe, No. 15, Clifford-Sreet. The Theory and Practice of Phyfic is delivered every day in the week, Sunday excepted, at eight o'clock in the morning; Chemiftry every Monday, Wednefday and Friday, at nine; and the Materia Medica, every Tuefday, Thurfday, and Saturday, at the fame hour.

---

Dr. Jenner, to whom the world is indebted for the introduction of the Variolæ Vaccinæ into the practice of Inoculation, has in the preſs new editions of his Treatiſes* on that important ſubject, with the addition of a copious appendix.

---

We underſtand, Mr. James Macartney is engaged in preparing for the preſs a tranſlation, accompanied with notes, of Prof. Soemmering's Syſtem of Anatomy now publiſhing in Germany, under the title, *De corporis humani fabrica,* a work univerſally admired, and poſſeſſing much original merit. Mr. M. alſo propoſes ſhortly to publiſh a ſmall work upon the Human Muſcles, the plan of which is in many reſpects new: it is deſigned to include the relation which each muſcle bears to other parts; and the combined operation of the muſcle in the functions and motions of the different parts of the body.

---

The fifth part of Mr. Charles Bell's Syſtem of Diſſections† (price 5s. 6d. each number; Johnſon, Longman and Rees) has lately appeared: it contains diſſections of the back part of the thigh, and of the leg and foot. We venture to ſay that this, when completed, will be a valuable work, both on account of the accuracy of the deſcriptions, and the fidelity of the engravings.

---

Dr. Batty will begin his Courſe of Lectures on the Theory and Practice of Midwifery, and on the Diſeaſes of Women and Children, on Monday, February 10th, at his houſe, No. 6, Great Marlborough-ſtreet, at half paſt ten o'clock in the morning.

---

Dr. Willich having, in one of the late Reviews, met with a malevolent and unwarrantable attempt to miſrepreſent the obvious tendency of his "Lectures on Diet and Regimen," he takes this opportunity of informing the Reviewer, whoſe name he underſtands is Dr. B****, that he is ready to prove ſatisfactorily, not only the fallacy of the inference drawn againſt the propriety of the Dedication, but alſo to demonſtrate the falſehood of the two other aſſertions contained in that abrupt criticiſm.

But as Dr. W. is determined to enter into no anonymous diſputes, which would violate the equitable principles adopted in *this* Journal, he requeſts that gentleman, to place himſelf firſt on an equal footing with thoſe whoſe works he has undertaken to cenſure; and he may reſt aſſured that Dr. W. on this occaſion, acts from no other motives than thoſe dictated by truth and juſtice; a duty which he owes to himſelf and the community.—*Medicus ratione utens nunquam alterum invidioſe calumniabitur; ſic enim animi impotentiam prodet.*

---

* Vid. our Journal No. I. p. 2, and No. IV. p. 313 and foll.

† Vid. our Journal, Vol. I. p. 404.

CRITICAL

